# Functional Brain Activation in Response to a Clinical Vestibular Test Correlates with Balance

**DOI:** 10.3389/fnsys.2017.00011

**Published:** 2017-03-10

**Authors:** Fatemeh Noohi, Catherine Kinnaird, Yiri DeDios, Igor S. Kofman, Scott Wood, Jacob Bloomberg, Ajitkumar Mulavara, Rachael Seidler

**Affiliations:** ^1^School of Kinesiology, University of MichiganAnn Arbor, MI, USA; ^2^Department of Psychology, University of MichiganAnn Arbor, MI, USA; ^3^Department of Mechanical Engineering, University of MichiganAnn Arbor, MI, USA; ^4^KBRwyleHouston, TX, USA; ^5^NASA Johnson Space CenterHouston, TX, USA

**Keywords:** VEMP, fMRI, skull tap, auditory tone burst, balance

## Abstract

The current study characterizes brain fMRI activation in response to two modes of vestibular stimulation: Skull tap and auditory tone burst. The auditory tone burst has been used in previous studies to elicit either a vestibulo-spinal reflex [saccular-mediated colic Vestibular Evoked Myogenic Potentials (cVEMP)], or an ocular muscle response [utricle-mediated ocular VEMP (oVEMP)]. Research suggests that the skull tap elicits both saccular and utricle-mediated VEMPs, while being faster and less irritating for subjects than the high decibel tones required to elicit VEMPs. However, it is not clear whether the skull tap and auditory tone burst elicit the same pattern of brain activity. Previous imaging studies have documented activity in the anterior and posterior insula, superior temporal gyrus, inferior parietal lobule, inferior frontal gyrus, and the anterior cingulate cortex in response to different modes of vestibular stimulation. Here we hypothesized that pneumatically powered skull taps would elicit a similar pattern of brain activity as shown in previous studies. Our results provide the first evidence of using pneumatically powered skull taps to elicit vestibular activity inside the MRI scanner. A conjunction analysis revealed that skull taps elicit overlapping activation with auditory tone bursts in the canonical vestibular cortical regions. Further, our postural control assessments revealed that greater amplitude of brain activation in response to vestibular stimulation was associated with better balance control for both techniques. Additionally, we found that skull taps elicit more robust vestibular activity compared to auditory tone bursts, with less reported aversive effects, highlighting the utility of this approach for future clinical and basic science research.

## Introduction

Recent studies have shown that a vestibular brain network is engaged when participants perform various motor and cognitive processes including sense of balance, postural control, navigation, spatial learning and memory, and autonomic responses to stress (Diener and Dichgans, 1988; Israel et al., 1996; Brandt et al., 2005; Smith et al., 2010; Guidetti, 2013; Hitier et al., 2014; Bigelow and Agrawal, 2015). The imaging of human vestibular cortex has opened a new window to our understanding of the underlying neural mechanisms of these motor and cognitive processes (Lobel et al., 1998; Fasold et al., 2002; Stephan et al., 2005). Neuroimaging meta-analyses have indicated the superior temporal gyrus (STG), retroinsular cortex, and inferior parietal lobule (IPL) as the common cortical regions involved in the vestibular network (Lopez et al., 2012; zu Eulenburg et al., 2012). Previous neuroimaging studies have used different methods to activate the vestibular cortex in the scanner, including caloric vestibular stimulation (CVS) (Bottini et al., 2001; Suzuki et al., 2001; Dieterich, 2003; Emri et al., 2003; Indovina, 2005; Marcelli et al., 2009), galvanic vestibular stimulation (GVS) (Lobel et al., 1998; Bense et al., 2001; Stephan et al., 2005; Eickhoff et al., 2006), and auditory clicks/short tone bursts (Miyamoto et al., 2007; Janzen et al., 2008; Schlindwein et al., 2008). In a recent meta-analysis, Lopez et al. (2012) showed that the regions that are commonly activated across these modes of stimulation include insular cortex, parietal operculum, and retroinsular cortex (Lopez et al., 2012).

Administration of CVS (i.e., irrigation of water into the ear canal) inside an MRI scanner is somewhat challenging, since the vestibular sensation takes up to 15 min to disappear (Proctor, 1988), and controlling for susceptibility artifacts between air and water is difficult (Lobel et al., 1996). Additionally, the evoked vestibular cortical activity by caloric stimulation varies based on the side and temperature of the irrigation (i.e., warm water induces bilateral brain activity, whereas cool water induces a contralateral response (Karim H. T. et al., 2013), and it shows high inter-individual variability (Fife et al., 2000). Moreover, caloric stimulation is rather unpleasant for subjects (Capps et al., 1973).

Galvanic vestibular stimulation is another common method in which electric current is used to stimulate the vestibular nerve via electrodes placed over the mastoid bones (Lobel et al., 1998; Yamamoto et al., 2005; Wuehr et al., 2016). GVS activates the entire vestibular nerve and thus activates pathways associated with both the semicircular canals and the otoliths, whereas CVS mainly activates the horizontal semicircular canals (Dieterich, 2003; Stephan et al., 2005; Day et al., 2011). Although GVS and CVS have been recently implemented as standard methods for imaging vestibular function, neither allows for evaluation of specifically otolithic vestibular processing (i.e., independent from semicircular canals).

Here, we evaluated a novel bone conduction device to elicit vestibular activation inside the MRI scanner (manufactured by Engineering Acoustics, Incorporated) (Iwasaki et al., 2008; Wackym et al., 2012). Curthoys et al. provided a review of animal and human studies in which they showed that the vestibular evoked myogenic potential (VEMP) characteristics in response to bone conduction stimulation reflects the otolith responses (utricles and saccules) independently of canal function (Curthoys et al., 2009). Brantberg et al. further supported these findings, showing that lateral bone conducted-vibrations induce VEMP responses through vibration and translation mechanisms (Brantberg et al., 2009).

Wackym et al. (2012) used bone conduction stimulation to elicit VEMPs, and then compared the response characteristics to those elicited by high decibel auditory tone bursts (Wackym et al., 2012). They showed the effectiveness of this novel device in eliciting typical vestibular responses. In the present study, we investigated whether the bone conduction method activates brain regions characterized in previous studies as playing a role in vestibular processing, and whether this activity correlates with vestibularly mediated balance control.

As Wackym et al. (2012) pointed out, the bone conduction method is more comfortable for subjects and results in more reliable oVEMP responses compared to auditory tone bursts (Wackym et al., 2012). In addition to subjects' comfort, this novel approach can provide new insights into vestibular utricular function (Curthoys et al., 2009; Manzari et al., 2010), since the previously used methods in neuroimaging studies have mainly indicated otolith responses with short auditory tone bursts (Schlindwein et al., 2008), semicircular canal responses with CVS (Gentine et al., 1990; Lobel et al., 1998; Fasold et al., 2002), or combined otolith and canal responses with GVS (Goldberg et al., 1984; Angelaki and Perachio, 1993; Stephan et al., 2005). Similar to GVS studies, a potential drawback of using the bone conduction method is tactile perception and potential co-activation of somatosensory regions (Eickhoff et al., 2006).

To replicate the results of Wackym et al. (2012), we used a similar design to compare vestibular activation elicited by skull taps (bone conduction) and auditory tone bursts both outside and inside the MRI scanner, hypothesizing that both stimulation methods would result in similar brain activation patterns. We also hypothesized that acoustic effects of the auditory tone burst stimulation could be differentiated from the vestibular components as previously done by Schlindwein et al. (2008).

To validate the vestibular evoked activation inside the scanner, we also recorded vestibular evoked myogenic potentials (VEMPs) elicited by the skull tap device and auditory tone bursts outside the scanner. The ocular vestibular evoked myogenic potential (oVEMP) has been measured in previous studies as an index of vestibular function (Akin et al., 2003). Therefore, we considered the brain activity as reflecting vestibular system activation relative to typical oVEMP characteristics (Welgampola and Colebatch, 2005; Nguyen et al., 2010) in response to the same stimulation outside of the scanner.

Further, we assessed individual differences in balance control ability to examine whether greater activation of vestibular network measured in the scanner is associated with better postural control measured outside of the scanner. Goble et al. (2011) showed that brain activity in response to proprioceptive stimulation of ankle joint muscles inside the scanner is correlated with individual differences in balance performance (Goble et al., 2011). Similarly, here we hypothesized that the magnitude of vestibular brain activity is associated with the ability to maintain balance, and that this association would be similar for both modes of stimulation. Using behavioral assessments we were able to fortify our interpretation of vestibular cortex function and to examine whether brain activation elicited by pneumatic skull tap and auditory tone burst associates differentially with behavioral metrics. Inline with our hypothesis, we found that the performance in balance control tasks (with degraded proprioceptive inputs and absence of vision) correlates with vestibular activity in right and left vestibular nuclei.

This study provides the first evidence of using an MR compatible pneumatic skull tap device to elicit vestibular brain activation inside the scanner. The results of this study indicate the extent to which pneumatic skull tap could be implemented in clinical and basic science research as a reliable method of vestibular otolith stimulation inside the scanner. Moreover, this study shows that vestibular brain activity elicited by pneumatic skull tap can be used as an index of individual differences in balance control and susceptibility to fall.

## Methods

### Participants

We recruited 16 healthy, right-handed young adults (mean age 20.87 ± 2.55, 7 females) from the University of Michigan student population. The study was approved by the University of Michigan Medical Institutional Review Board; all participants signed a consent form prior to participation. Exclusion criteria comprised history of neurological disorder, vestibular, or auditory impairments, or any other major health issues. All participants were right hand dominant. Two participants were excluded from analyses because of incomplete MRI scan coverage of the brain. From the remaining 14 participants who were included in the fMRI analysis, only 10 were included in the balance analyses due to substandard quality of the force plate data.

### Balance assessments

To assess whether individual differences in balance control are associated with vestibular brain activity, subjects performed four different tasks (Romberg, tandem, normal, and single leg stance) in four different levels of difficulty (eyes closed/open, yaw/pitch/no head movement, arms crossed/free, & on firm/compliant surface). These tests were conducted on a force platform (AMTI Inc, USA) and subjects' movements were captured with a Vicon motion capture system (Nexus, Vicon Inc). We selected two balance tasks for the brain-behavior correlation analyses: Romberg stance (feet together) with eyes closed and sinusoidal head movements (roughly ±20°, 0.6 Hz); and Tandem Romberg stance (heel to toe) on a compliant surface (high density viscoelastic foam; length = 45 cm, width = 45 cm, thickness = 18 cm; Natus Inc.), with eyes open and sinusoidal head movements (roughly ±20°, 0.6 Hz). The tasks were performed once with yaw and once with pitch head movements. We instructed subjects to match their head movements to the beat of a metronome (0.6 Hz) to keep the frequency of movement consistent throughout the trials and across subjects. Subjects maintained a comfortable amplitude with head turns of ~20°, however this amplitude was not strictly controlled. The order of tasks was counterbalanced across subjects.

Subjects were instructed to attempt to maintain their balance for 30 s for each task. The experimenter demonstrated the correct performance prior to each trial; however, to capture the individuals' true postural ability and control for the learning effects, they were not given any practice trials. The total amount of movement was calculated as the area of an ellipse fit to the 95th percentile confidence interval of center of pressure motion in the anterior-posterior and medial-lateral directions (Lee et al., 2012). Smaller ellipse areas reflect smaller body sway and better performance. Ellipse areas and balance maintenance times were tested for correlation with vestibular brain activity by entering scores as covariates in a single sample *t*-test analysis of vestibular activity, using spm8 software (Wellcome Department of Cognitive Neurology, London, UK; Friston et al., 1995).

### Ocular vestibular evoked myogenic potentials (oVEMP)

Prior to the MRI scan, we measured vestibular evoked myogenic potentials in ocular muscles in response to both auditory tone bursts and head taps applied outside of the MRI scanner (cf., Todd et al., 2007; Iwasaki et al., 2008). First, we performed routine skin preparation using alcohol wipes and Nuprep skin preparation gel. Next, EMG electrodes were placed symmetrically on the skin over the medial inferior oblique muscles, slightly lateral to the pupil directly beneath both eyes (Chihara et al., 2009). We collected ocular EMG data at 1 KHz using a Delsys 8-channel Bagnoli EMG system. Subjects were in a supine position, maintaining an ~30° upward gaze during the stimulation trials by staring at a fixation point placed on the ceiling. They were instructed to avoid blinking for the duration of each stimulation trial (tap stimulation trials lasted 29 s, while tone stimulation trials lasted 20 s; first 5 s in both trials were baseline when no stimulation applied), and to relax their eyes in between trials. Subjects received auditory tone bursts (~3 Hz) via headphones (MR compatible SereneSound auditory system, Resonance technology Inc.) and skull taps (1 Hz) via the pneumatic tactile pulse system [MR compatible Pneumatic Tactile Pulse System (PnTPS), Engineering Acoustics Inc.; see Supplementary Material Figure 1] placed over the lateral cheekbones. The lateral direction of the force of the skull tap served to maximize the shear along the utricular macula, and therefore optimize the oVEMP response that is attributed to utricular-ocular reflex pathway (Curthoys et al., 2012). A self-adhering elastic bandage wrap (Coban, 3 M Inc) was used over the subject's head to secure the stimulation devices. We collected five trials of each stimulation type for each subject and visually inspected the data after each trial and adjusted the tapper/headphones as necessary.

Each subject received auditory tone burst stimulation at 130 dB SPL on the left side (five trials) and right side (five trials) separately. A pneumatically powered skull tapper was used to deliver low force taps to the left (five trials) and right (five trials) cheekbones (Engineering Acoustics Inc). We applied unilateral stimulation since Cornell et al. (2009) reported that bilateral bone conduction vibration activates deeper muscles and eliminates the horizontal eye response (Cornell et al., 2009). The skull tapper uses compressed air (50–55 psi) to power a small piston that delivers an average force of 19.6 N for each tap to the cheekbones. Each tapping trial consisted of 24 taps, delivered at 1 Hz. Each auditory tone burst trial consisted of 45 tone bursts applied at 3.003 Hz. The order of stimulation modes was counterbalanced across subjects. The resting period between trials varied based on the subject's preference, ranging between 10 and 15 s. Stimulation routines were programmed using LabView (National Instruments Inc.).

### Functional magnetic resonance imaging (fMRI)

fMRI data were acquired using a 3.0 T MRI scanner (General Electric Medical Systems, DISCOVERY MR750). Using a self-adhering elastic bandage wrap, the experimenter fixed the position of the pneumatic tappers and auditory headphones before the subject entered the scanner. To keep consistent placement of devices, we conducted the oVEMP measurements prior to the scan and did not change the device arrangement (i.e., placement of tappers on the cheekbones) as we proceeded with the scan. Head movement was minimized via a Velcro strap over the forehead and padding placed around the sides of the head. Subjects' physiological responses were collected via pulse oximeter placed on the index finger, and a respirometer wrapped around the subjects' abdomen, and later regressed out of the blood oxygenation level dependent (BOLD) signal.

The scan protocol comprised five sections: A high resolution T1 scan, a resting state functional connectivity scan, vestibular stimulation runs (with auditory and tapper trials counterbalanced across subjects), another resting state functional connectivity scan, and a diffusion tensor scan (DTI). The resting state and DTI scan results are not presented here. Before each section began, the MRI technician notified the subject about the upcoming condition. This way we minimized the potential artifacts of surprise (e.g., involuntary responses to the start of the stimulation) and kept the subject alert.

The structural imaging was conducted using a T1-weighted interleaved echo-planar imaging (EPI) sequence (TR = 12.2 s, TE = 5.1 ms, FA = 15°, matrix size = 256 × 256, FOV = 260 × 260 mm, slice thickness = 1 mm) covering the whole brain and the cerebellum. The functional images were acquired with gradient-echo spiral-pulse sequence (FOV = 220 mm, TR = 2 s, TE = 30 ms, number of slices = 43, voxel size = 3.4375 × 3.4375 mm).

The skull tap and auditory tone burst stimulations were applied using the same protocol as was used for oVEMP testing outside of the scanner, with two exceptions: (1) following the auditory tone burst stimulation (130 dB SPL) on the left and right sides separately, there was an additional condition in which subjects received 90 dB SPL auditory tone burst stimulation on both sides simultaneously. By including the 90 dB SPL stimulation we were able to compare and dissociate the neural correlates of acoustic and vestibular processing (Schlindwein et al., 2008); (2) Unlike in the oVEMP testing, there was no subject-determined rest period between stimulation trials. Rather, to identify stimulation-evoked changes in the BOLD signal we implemented a block design in which each functional run comprised five alternating periods of rest (20 s) and stimulation (24 s). Each run was 4 min, and subjects were asked to keep their eyes closed during each run. The sound of the piston delivering taps to the cheekbones was not detectable over the noise of the scanner, and there was no head motion induced by the taps. All subjects reported feeling a tactile perception of the pneumatic skull taps over the skin.

## Data analysis

### Balance performance

Force plate data were collected at 100 Hz and center of pressure (COP) values were measured analyzed using Vicon software (Nexus, Vicon Inc). The COP signals were low pass filtered with a 2nd order recursive Butterworth filter with a cutoff of 10 Hz (Lee et al., 2012) using Matlab. Subsequently, 95% confidence interval ellipses were fit to the 2 dimensional medial-lateral and anterior-posterior center of pressure trajectories across each 30 s balance task. In case of a step out, the trial was stopped prematurely, and the balance maintenance time was used as a second representative of subjects' balance ability in addition to the measures of body sway.

### oVEMP

We identified the oVEMP EMG response according to the typical waveform described in previous studies (Welgampola and Colebatch, 2005; Nguyen et al., 2010). We parsed the data into responses for each individual tap (from tap-to-tap), and the oVEMPs were averaged on the contralateral stimulation sides. We identified the amplitude and timing of the first peak, followed by the first trough, followed by the second peak of the VEMPs. We used a 2nd order butterworth notch filter to remove 60 Hz noise (59–61 Hz) and the detrend function in Matlab to remove the mean value from the vector. We did all of the above for the auditory tone burst induced oVEMPs as well, but added some additional notch filters for harmonics of electrical noise (harmonics of 60 Hz): 60, 120, 240, 360 Hz.

### fMRI data analyses

The fMRI preprocessing analyses were conducted using spm8 software (Welcome Department of Cognitive Neurology, London, UK; Friston et al., 1995). The first 10 volumes in each run were discarded to ensure steady state of the MR signal at the beginning of the runs. Functional data were corrected for the physiological responses (i.e., cardiac and respiration data) using the RETROICOR algorithm (Glover et al., 2000). Since the skull vibration induced by the pneumatic taps could be a potential source of motion artifacts during EPI acquisition, the raw data were carefully examined for excessive motion. Head motion correction was implemented; the cut off for trial exclusion was >3 mm translation or >5° rotation. The functional images were realigned to the first functional image and the anatomical image. Next, both functional and anatomical images were normalized to the Montreal Neurological Institute (MNI) template (Friston et al., 1995). We normalized the cerebellum to the Spatially Unbiased Atlas Template (SUIT, Diedrichsen, 2006; Diedrichsen et al., 2009, 2011; Diedrichsen and Zotow, 2015). Caret software (Van Essen et al., 2001) was used for hand corrected isolation of the cerebellum and brainstem from the brain. These isolated cerebellar images were subsequently analyzed and are presented separately from the whole brain results. The normalized functional images were spatially smoothed with a Gaussian kernel function (8,8,8 mm). The smoothed functional images were used to design the first level analysis, in which we compared brain activity in each condition to rest. Next, we used the contrast images (created at the single subject level) for whole group analyses. We applied one-sample *t*-tests to measure brain activation and deactivation in stimulation trials compared to rest across all subjects. Further, we applied paired *t*-tests to compare brain activity between different stimulation conditions across all subjects. We applied a threshold of *P* ≤ 0.001 (unc.) with a minimum cluster size of 10 voxels (voxel size = 2 × 2 × 2 mm) for all contrasts. To find the common brain regions activated by both auditory tone bursts and pneumatic taps, we conducted a conjunction analysis across these two conditions. The threshold for conjunction analyses was determined based on p^1/n^, where p is the individual threshold and *n* is the number of contrasts in the conjunction (Friston et al., 1999).

We assessed the correlation between brain activity and balance control using the balance performance parameters (i.e., area of the ellipse and balance maintenance time) as covariates in our group analyses, with a threshold of *p* < 0.001 (unc.). The correlation analyses were limited to the identified brain regions in the previous step.

We used the MNI atlas (Friston et al., 1995) to localize the significant coordinates resulting from our analyses. The cerebellar coordinates were localized according to the SUIT atlas (Diedrichsen et al., 2009) using MRIcron. We also applied Automated Anatomical Labeling (AAL, Tzourio-Mazoyer et al., 2002) with a gray matter inclusive mask to filter out activity in the white matter. A small volume correction was also applied to examine activity in regions previously identified as the vestibular nuclei (*x* = −16/16, *y* = −36, *z* = −32) (Kirsch et al., 2015). Deep cerebellar nuclei were identified using the SUIT probabilistic atlas for deep cerebellar nuclei (Diedrichsen et al., 2011).

Finally, to provide an estimate of the effect size, we used the SPM MarsBaR toolbox to extract the beta values for each significant cluster. We did not report the percent signal change since previous studies showed that MR acquisition parameters (e.g., field strength, scanner sequence, echo time, etc.) could influence this metric (Uludaǧ et al., 2009; Chen et al., 2016). Thus, we reported the beta values for each significant cluster. First, we defined the region of interest (ROI) as a 5 mm sphere around the coordinates at the peak value. Next, we reported the mean beta values averaged across the voxels within each ROI (Kong et al., 2007; Hein et al., 2010; Oechslin et al., 2010).

## Results

### Balance performance

**Figure 1:**
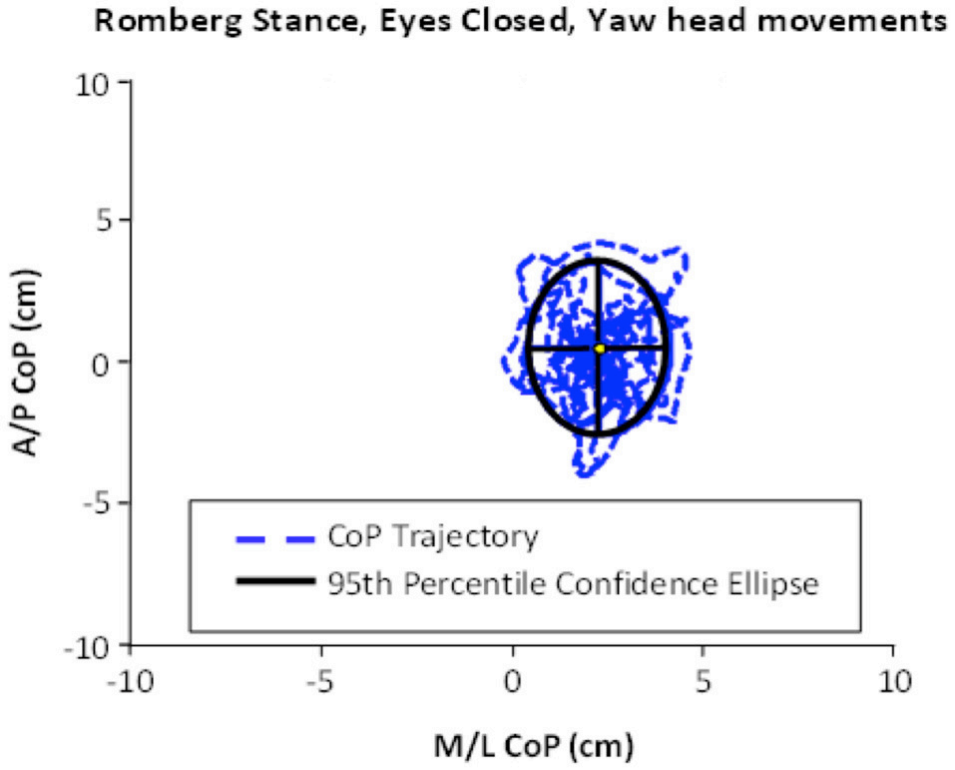
**An example of ellipse area, calculated based on the center of pressure (CoP) motion in the anterior–posterior (A/P) and medial-lateral (M/L) trajectories**. This example shows the performance of Romberg stance on firm stance, with eyes closed and yaw head movement.

As shown in Table 1, all subjects were able to maintain their balance for 30 s when performing the Romberg stance with eyes closed and making yaw or pitch head movements. The tandem stance on a compliant surface with eyes open and yaw/pitch head movements was found to be more difficult, as the average balance time in this task did not reach the 30 s cap. In addition, the average amount of body sway was greater in tandem conditions compared to Romberg (see Table 1 for details). An example of body sway trajectories (captured in an ellipse) is presented in Figure 1.

**Table 1:** **List of balance tasks that subjects performed on the force platform**.

**Balance tasks**	**Time _Seconds_**	**Ellipse area _cm^2^_**
Romberg stance, eyes closed, firm surface, yaw head movement	30 (0)	8.36 (4.14)
Romberg stance, eyes closed, firm surface, pitch head movement	30 (0)	12.84 (9.39)
Tandem stance, eyes open, compliant surface, yaw head movements	20.05 (12.0)	39.60 (32.53)
Tandem stance, eyes open, compliant surface, pitch head movements	28.11 (3.95)	29.23 (20.25)

*Mean and standard deviation (in parenthesis) of balance maintenance times and ellipse areas are shown for each task*.

To assess the correlation between balance and vestibularly mediated brain activity, we used the ellipse area measurements of “Romberg stance on firm surface, with eyes closed and yaw/pitch head movements”, because the time factor in performing this task was equal for all subjects (see Correlation between brain activity and balance. below). Only 10 subjects were included in this analysis due to substandard quality of force plate data for the remaining four subjects. In addition, we used the time measurements of “tandem stance on a compliant surface with eyes open and yaw/pitch head movements”, because not all subjects were able to maintain their balance for 30 s when performing this task.

### Ocular VEMP

**Figure 2:**
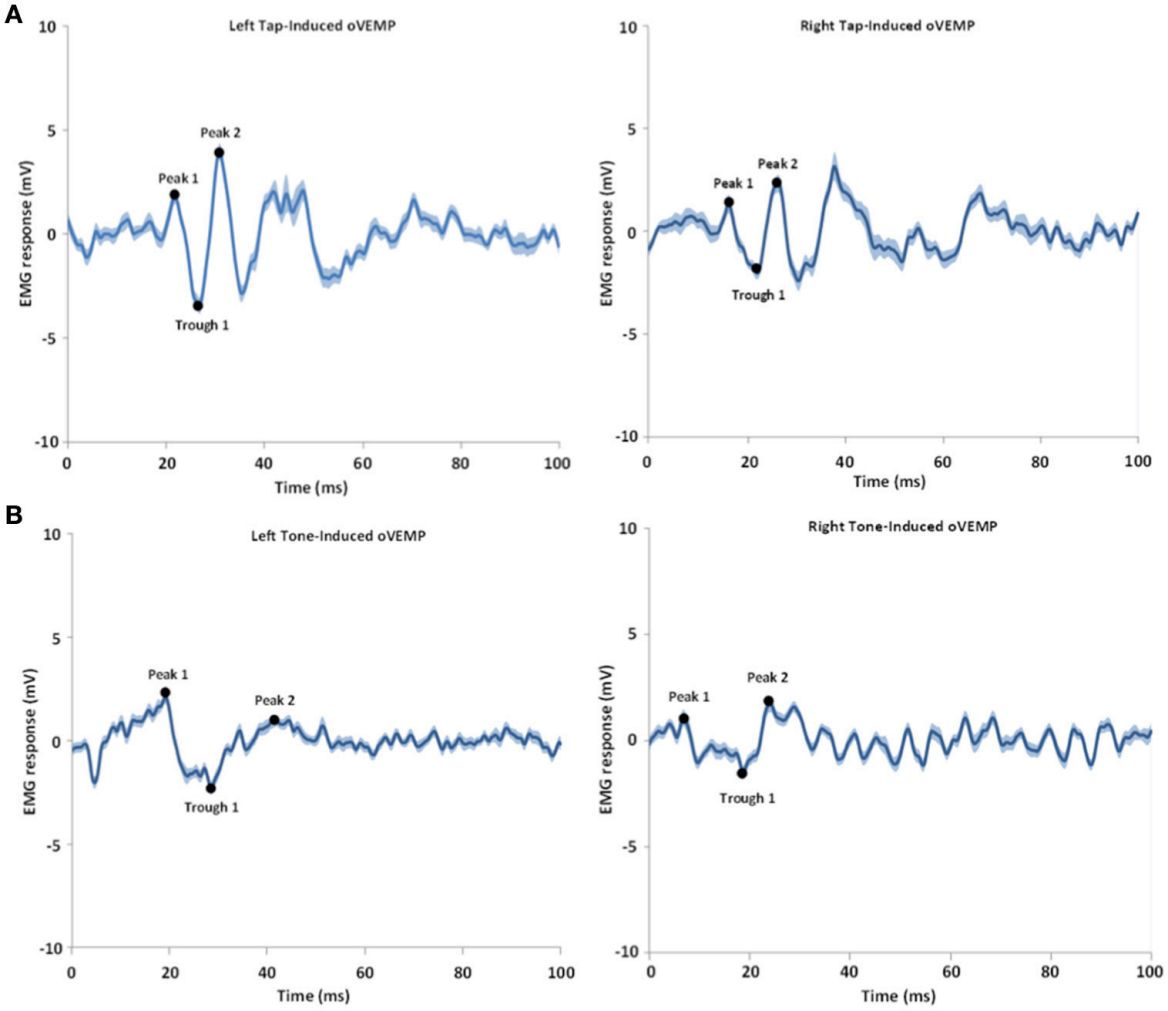
**Shows an example of tap-induced (A)** and tone-induced **(B)** oVEMPs for one subject. The left and right refer to the left and right sides of stimulation; the oVEMPs represent the averaged response on the contralateral side of stimulus delivery. The shaded areas represent the standard error of the mean (SEM). Time 0 indicates the stimulus onset time.

As shown in Table 2 and Figure 2, the overall oVEMP characteristics of our data fit the typical oVEMP response (Iwasaki et al., 2008; Todd, 2010; Wackym et al., 2012), which validates the observed vestibular-evoked activation inside the scanner. There was no significant effect of side/mode of the stimulation on the elicited oVEMP characteristics.

**Table 2:** **Shows the mean and standard error (in parenthesis) of oVEMPs**.

**oVEMP characteristics**	**Tap-induced response**		**Tone-induced response**	
	**Left tap**	**Right tap**	**Left tone**	**Right tone**
Peak 1 latency _ms_	13.43 (2.04)	14.95 (2.01)	9.82 (2.36)	12.1 (2.65)
Trough 1 latency _ms_	17.63 (2.10)	19.83 (2.14)	15.19 (2.76)	16.77 (2.79)
Peak 2 latency _ms_	23.34 (2.06)	25.01 (2.34)	24.89 (3.40)	23.3 (2.87)
Peak to Peak Amplitude 1_mv_	3.91 (0.55)	4.36 (1.08)	3.27 (0.72)	2.65 (0.59)
Peak to Peak Amplitude 2_mv_	5.39 (1.05)	5.59 (1.35)	3.26 (0.99)	2.86 (0.41)

*The left and right refer to the side of the stimulation, and oVEMPS are the averaged response on the contralateral side (i.e., left tap column represents the right infraorbital eye muscle's response to left side tap stimulation). The latencies of tap-induced responses are corrected for hardware delay. The latencies are presented in milliseconds and amplitudes are in millivolts. Peak to peak amplitude1 = change between the amplitude at first peak to the first trough; peak to peak amplitude2 = change between the amplitude at first trough to the second peak*.

### fMRI results

#### Tap stimulation vs. rest

The results for tap-induced alterations in brain function are presented in Table 3.

**Table 3:** **Results of activation and deactivation for left and right tap vs. rest**.

***t*-contrast**	**Brain region**	**MNI coordinates x,y,z**	**Cluster size**	***t*-value**	***P*-value**	**β (CI)**
**LEFT TAP VS. REST**						
Activation	Right Superior Temporal Gyrus	58, −30, 14	1118	5.79	0.0002^*^	0.65 (±0.23)
	Left Posterior Insula	−50, −32, 20	222	5.56	0.0006^*^	0.37 (±0.14)
	Right Posterior Insula	38, −12, 16	12	3.61	0.0008	0.14 (±0.07)
Deactivation	Left Fusiform Gyrus	−30, −40, −18	189	4.55	0.0004	−0.15 (±0.05)
	Right Cerebellar Lobule VI	30, −44, −35	152	5.21	0.0001	−0.09 (±0.04)
	Left Cerebellar Lobule VI	−32, −46, −31	37	4.40	0.0001	−0.12 (±0.05)
	Left Precuneus	−22, −84, 44	125	4.56	0.0002	−0.20 (±0.07)
	Right Precuneus	8, −52, 60	93	3.57	0.0004	−0.15 (±0.07)
	Left Middle Frontal Gyrus	−36, 42, 34	39	3.71	0.0004	−0.17 (±0.08)
	Right Inferior Temporal Gyrus	56, −8, −14	21	3.75	0.0004	−0.17 (±0.09)
	Right Inferior Frontal Gyrus	28, 30, −4	21	3.08	0.0008	−0.09 (±0.04)
	Right Fusiform Gyrus	34, −36, −16	16	3.43	0.0008	−0.06 (±0.03)
	Right Middle Frontal Gyrus	22, −12, 58	12	3.12	0.0008	−0.08 (±0.04)
	Left Superior Frontal Gyrus	−20, 52, −4	10	3.43	0.0008	−0.11 (±0.06)
	Right Brainstem, Pons	6, −26, −15	189	4.75	0.0001	−0.08 (±0.05)
	Left Brainstem, Pons	−6, −28, −9	189	6.15	0.0001	−0.13 (±0.07)
	Left Cerebellar Lobule VIIIA	−2, −60, −31	29	4.29	0.0001	−0.05 (0.04)
**RIGHT TAP VS. REST**						
Activation	Left Posterior Insula	−52, −34, 18	682	5.91	0.0002	0.41 (±0.13)
	Left Postcentral Gyrus	−54, −12, 14	682	4.38	0.0002	0.25 (±0.12)
	Right Superior Temporal Gyrus	60, −32, 16	296	5.13	0.0004	0.42 (±0.16)
	Right Posterior Insula	50, −36, 20	296	4.57	0.0002	0.36 (±0.17)
Deactivation	Left Cerebellar Crus I	−36, −54, −35	640	7.63	0.0001^*^	−0.09 (±0.03)
	Left Cerebellar Lobule VI	−10, −64, −27	144	5.11	0.0001	−0.06 (±0.04)
	Right Cerebellar Lobule VIIIA	4, −66, −33	144	3.99	0.0010	−0.03 (±0.03)
	Left Temporal Lobe, Sub-Gyral	−42, −46, −6	96	6.21	0.0002	−0.09 (±0.02)
	Left Cingulate Gyrus	−16, −2, 48	50	5.28	0.0004	−0.08 (±0.03)
	Right Thalamus	4, −6, 2	30	5.63	0.0006	−0.10 (±0.04)
	Right Paracentral Lobule	16, −40, 50	17	4.93	0.0004	−0.07 (±0.03)
	Left Vestibular Nucleus	−22, −44, −31	17	4.89	0.0001^*^	−0.04 (±0.04)

* *Significant at FWE. P < 0.05. Mean beta values (β) are represented with %95 confidence interval (CI)*.

**Figure 3:**
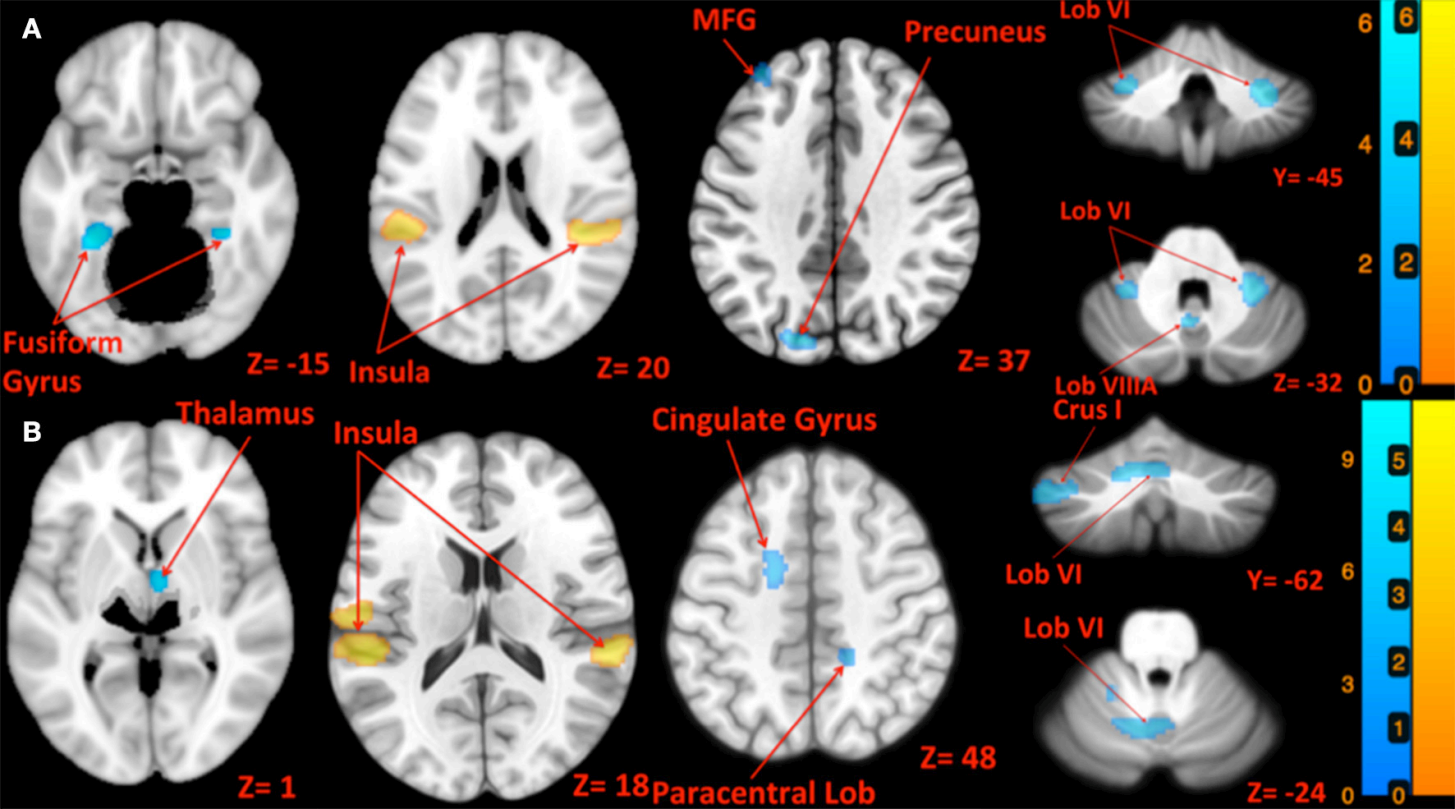
**Map of brain activation and deactivation for left [A:** Left tap > rest (yellow); Left tap < rest (blue)] and right [**B**: Right tap > rest (yellow); Right tap < rest (blue)] taps. The corresponding t-value is presented next to each figure. Left and right sides refer to the left and right sides of the brain, respectively. Note: the cerebellum is depicted separately due to extraction and normalization to the SUIT template. N.S, Non-Significant; MFG, Middle Frontal Gyrus.

Left side tap stimulation increased the activation of right STG and bilateral posterior insula (Figure 3A), both portions of the canonical vestibular cortex (zu Eulenburg et al., 2012). Multiple regions exhibited deactivation in response to the left tap, including bilateral cerebellum lobule VI, left cerebellum lobule VIIIA, bilateral brainstem (pons), bilateral precuneus, bilateral fusiform gyrus, and bilateral frontal regions including superior, middle and inferior frontal gyri (Figure 3A).

Right side tap stimulation activated the bilateral posterior insula, left postcentral gyrus, and right STG (Figure 3B). Similar to the left tap deactivation pattern, the right tap deactivated multiple regions including left cerebellum lobule VI and Crus I, left vestibular nucleus, right cerebellum lobule VIIIA, left temporal lobe sub gyral, left cingulate gyrus, right paracentral lobule, and the right thalamus (Figure 3B).

#### Auditory tone burst stimulation vs. rest

The results for tone-induced alterations in brain function are presented in Table 4.

**Table 4:** **Results of activation and deactivation for left, right, and both sides tone stimulation vs. rest**.

***t*-contrast**	**Brain region**	**MNI coordinates x,y,z**	**Cluster size**	***t*-value**	***P*-value**	**β (CI)**
**LEFT TONE (130 dB) VS. REST**						
Activation	Right Superior Temporal Gyrus	58, −38, 12	614	5.19	0.0002	0.51 (±0.20)
	Left Middle Frontal Gyrus	−44, 26, 16	198	6.76	0.0002	0.11 (±0.03)
	Left Middle Frontal Gyrus	−36, 12, 30	170	4.82	0.0002	0.07 (±0.03)
	Left Superior Temporal Gyrus	−44, −28, 8	152	4.45	0.0006	0.25 (±0.11)
	Left Middle Temporal Gyrus	−68, −40, −8	127	5.72	0.0006	0.16 (±0.05)
	Left Superior Temporal Gyrus	−64, −24, 8	44	4.06	0.0008	0.56 (±0.27)
	Left Precentral Gyrus	−54, −4, 54	17	4.50	0.0008	0.21 (±0.11)
	Left Caudate	−18, 16, 12	13	5.54	0.0002	0.06 (±0.02)
Deactivation	No suprathreshold voxels were found					
**RIGHT TONE (130 dB) VS. REST**						
Activation	No suprathreshold voxels were found					
Deactivation	Right Cuneus	4, −68, 30	82	5.89	0.0002	−0.13 (±0.04)
	Right Posterior Cingulate	4, −48, 18	40	8.38	0.0002	−0.07 (±0.01)
	Left Insula	−38, −2, 20	19	4.19	0.0004	−0.05 (±0.02)
	Right Precuneus	16, −48, 42	18	4.18	0.0004	−0.05 (±0.02)
**BOTH SIDES TONE (90 dB) VS**. **REST**						
Activation	Left Middle Temporal Gyrus	−70, −14, −18	315	7.67	0.0002^*^	0.14 (±0.05)
	Left Middle Frontal Gyrus	−30, 24, 58	236	7.14	0.0002^*^	0.15 (±0.04)
	Left Inferior Parietal Lobule	−52, −52, 54	163	6.03	0.0002	0.20 (±0.10)
	Left Superior Frontal Gyrus	−4, 28, 60	51	6.24	0.0002	0.18 (±0.06)
	Left Insula	−36, 8, 20	17	5.83	0.0004	0.04 (±0.01)
	Left Superior Frontal Gyrus	−14, 46, 30	15	4.74	0.0004	0.10 (±0.04)
	Right insula	36, 10, 20	27	4.47	0.0001	0.09 (±0.04)
Deactivation	Left Parahippocampal Gyrus	−40, −44, 2	17	4.47	0.0004	−0.05 (±0.03)
	Left Temporal Lobe, Sub-Gyral	−42, −40, −6	17	3.53	0.0006	−0.04 (±0.02)

* *Significant at FWE. P < 0.05. Mean beta values (β) are represented with %95 confidence interval (CI)*.

**Figure 4:**
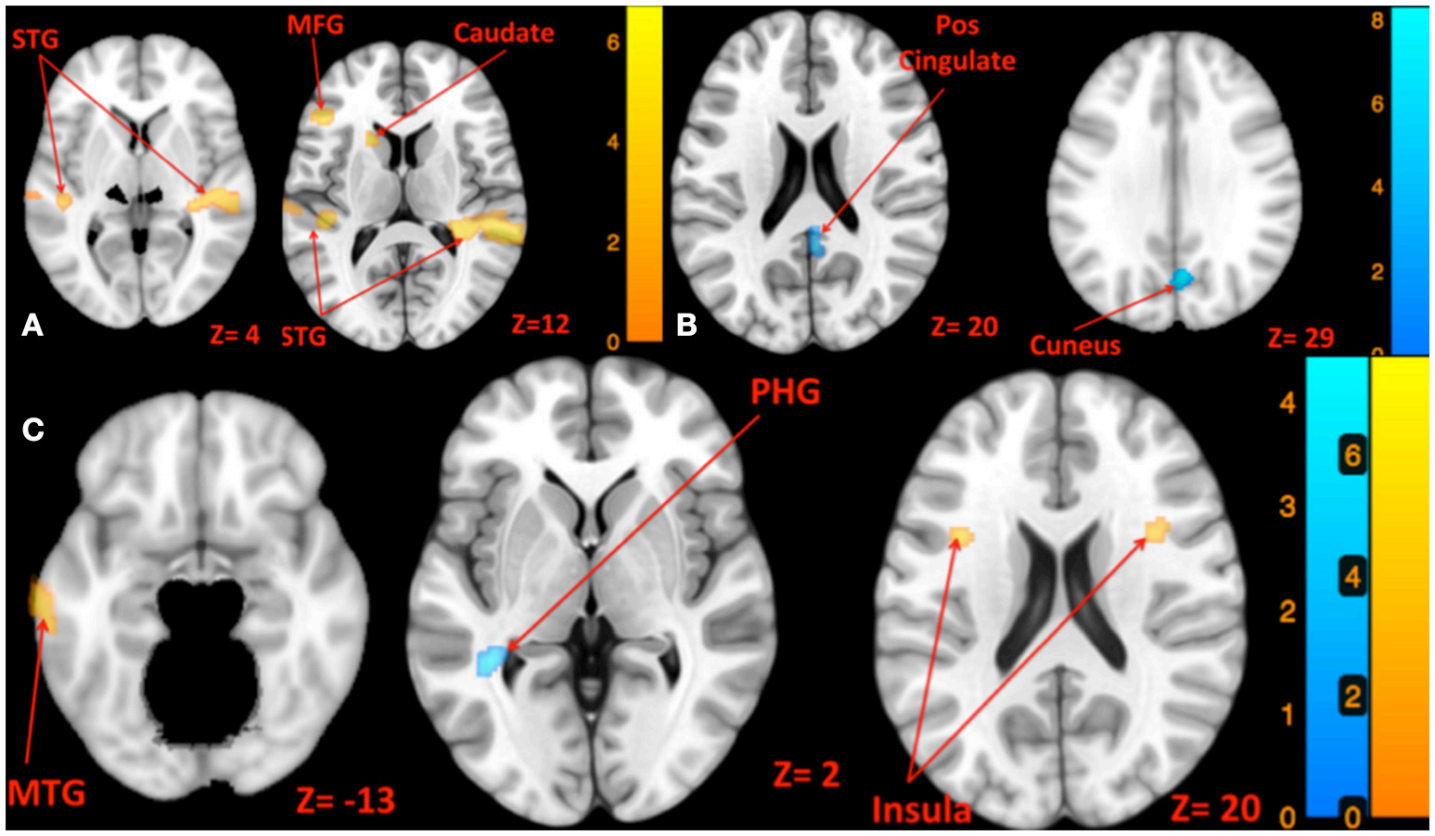
**Map of brain activation and deactivation for left [A:** Left tone > rest (yellow); Left tone < rest (N.S)], right [**B**: Right tone > rest (N.S); Right tone < rest (blue)], and both sides [**C**: Both side tone > rest (yellow); Both side tone < rest (blue)] tone stimulation. The corresponding *t*-value is presented next to each figure. Left and right sides refer to the left and right sides of the brain, respectively. N.S, Non-Significant; STG, Superior Temporal Gyrus; MFG, Middle Frontal Gyrus; MTG, Middle Temporal Gyrus; PHG, Parahippocampal Gyrus.

Left side tone stimulation (130 dB SPL) mainly activated the right STG. Additionally, it resulted in activation of ipsilateral regions including the left middle frontal and precentral gyrus, left superior and middle temporal gyrus, and left caudate (Figure 4A). Left tone stimulation did not result in significant deactivation.

Right side tone stimulation (130 dB SPL) did not result in a significant increase in brain activity compared to rest. However, it deactivated multiple regions including right cuneus, right posterior cingulate, right precuneus, and left insula (Figure 4B).

Both sides tone stimulation (90 dB SPL) resulted in activation of multiple regions predominantly in the left hemisphere including the middle temporal gyrus, middle and superior frontal gyri, left inferior parietal lobule, and bilateral activation of the insula (Figure 4C). There was a unilateral pattern of deactivation including left parahippocampal gyrus and left temporal lobe sub-gyral (Figure 4C).

#### Conjunction results

**Figure 5:**
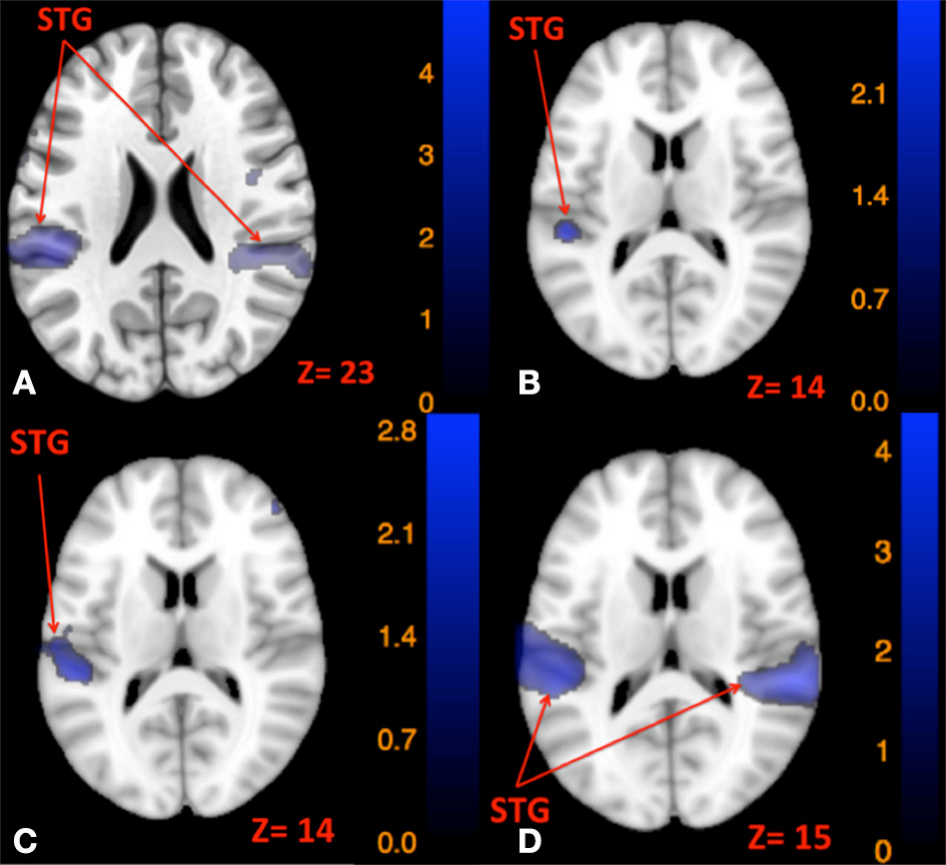
**Map of brain regions commonly activated by left tap and left tone stimuli (A)**, right tap and right tone stimuli **(B)**, left tap and right tone stimuli **(C)**, and right tap and left tone stimuli **(D)**. The corresponding *t*-value is presented next to each figure. Left and right sides refer to the left and right sides of the brain, respectively. N.S, Non-Significant; STG, Superior Temporal Gyrus.

Using conjunction analyses we were able to locate common regions activated by tap and tone stimulation modes (Table 5). The results showed that left side tap and left side tone stimulations commonly activated the right and left STG (Figure 5A), whereas right side tap and right side tone stimulations commonly activated the left STG (Figure 5B).

**Table 5:** **Conjunction results for commonly activated regions by tap and tone stimuli**.

***t*-contrast**	**Brain region**	**MNI coordinates x,y,z**	**Cluster size**	***t*-value**	***P*-value**	**β (CI)_tap**	**β (CI)_tone**
**LEFT TAP AND LEFT TONE**							
	Right Superior Temporal Gyrus	58, −38, 12	39	4.97	0.0001	0.48 (±0.18)	0.51 (±0.20)
	Left Superior Temporal Gyrus	−48, −34, 20	1101	4.33	0.0001	0.32 (±0.12)	0.32 (±0.16)
**RIGHT TAP AND RIGHT TONE**							
	Left Superior Temporal Gyrus	−50, −32, 14	48	2.78	0.005	0.35 (±0.14)	0.19 (±0.15)
**LEFT TAP AND RIGHT TONE**							
	Left Superior Temporal Gyrus	−52, −32, 14	238	2.92	0.004	0.34 (±0.18)	0.20 (±0.15)
	Right Inferior Parietal Lobule	48, −48, 50	16	2.26	0.016	0.15 (±0.12)	0.10 (±0.10)
**RIGHT TAP AND LEFT TONE**							
	Left Insula	−50, −36, 18	1525	4.37	0.0001	0.31 (±0.11)	0.33 (±0.15)
	Left Superior Temporal Gyrus	−62, −22, 12	1525	3.52	0.0010	0.49 (±0.28)	0.58 (±0.31)
	Right Superior Temporal Gyrus	58, −36, 14	1139	4.43	0.0001	0.43 (±0.19)	0.55 (±0.23)
	Left Precentral Gyrus	−54, −4, 54	78	3.51	0.0010	0.18 (±0.15)	0.21 (±0.11)

*Mean beta values (β) are represented with %95 confidence interval (CI)*.

Since the primary contrasts between stimulation modes and rest revealed a potential laterality effect, we conducted additional conjunction analyses to locate possible common regions activated by the two stimulation modes delivered to opposite sides: Left side tap and right side tone commonly activated the left STG (Figure 5C); whereas right side tap and left side tone commonly activated the left insula, left STG, and right STG (Figure 5D).

#### Correlation between brain activity and balance

**Figure 6:**
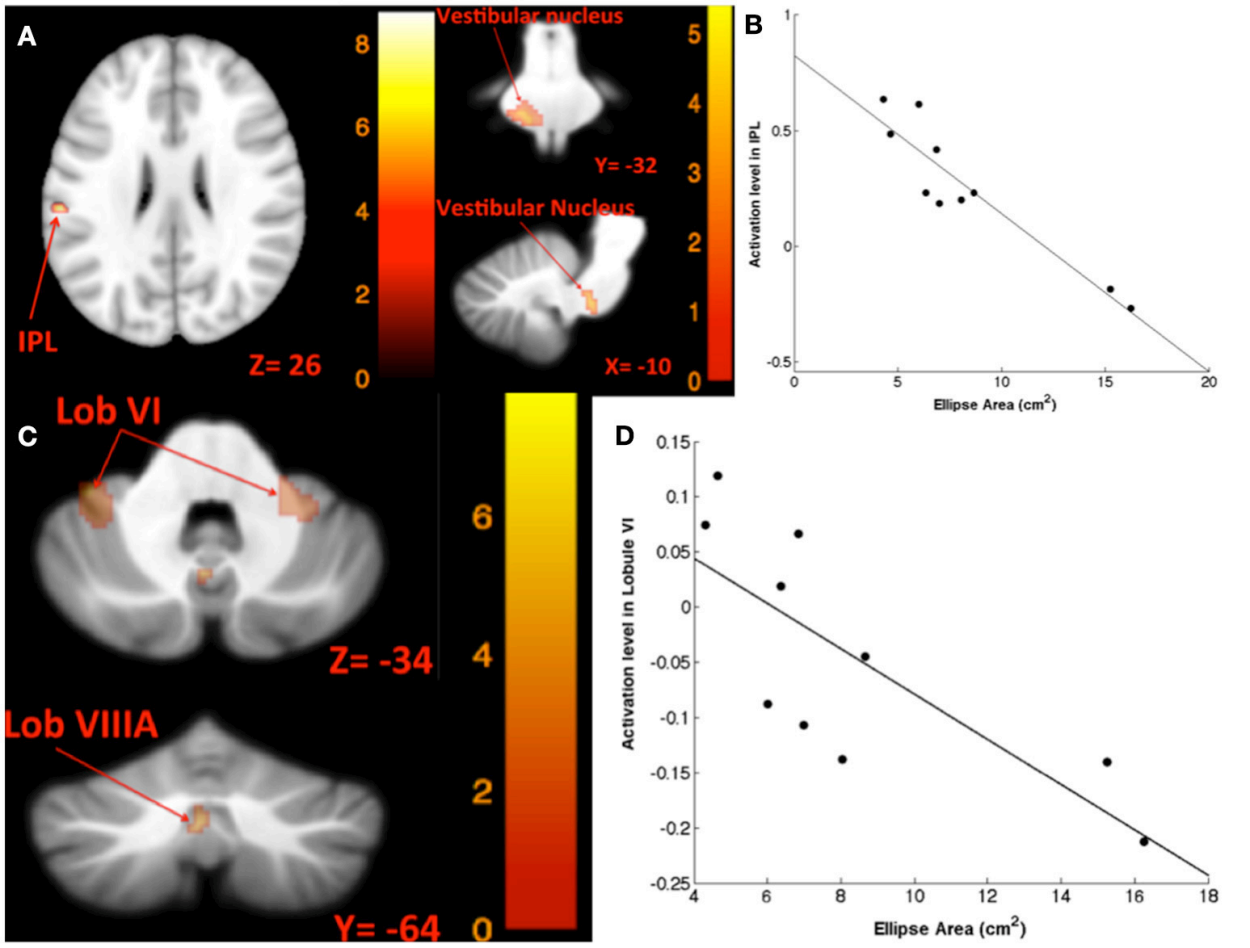
**Map of correlation between ellipse area and brain activity induced by left tap**. Left tap correlation with ellipse area in Romberg stance on firm surface, eyes closed, yaw head movement **(A)**; the scatter plot illustrates the correlation between ellipse area and activation in IPL, suggesting that greater activation in IPL was correlated with smaller ellipse area **(B)**; left tap correlation with ellipse area in Romberg stance on firm surface, eyes closed, pitch head movement **(C)**; the scatter plot illustrates the direction of correlation between ellipse area and activation in cerebellar lobule VI, suggesting that less deactivation of cerebellar lobule VI is correlated with smaller ellipse area **(D)**. The corresponding *t*-value is presented next to each figure. Left and right sides refer to the left and right sides of the brain, respectively. N.S, Non-Significant; IPL, Inferior Parietal Lobule.

The results for correlation between tap-induced brain activity and ellipse area are presented in Table 6. There was a significant correlation between left side tap-induced brain activity and body sway (in Romberg stance on firm surface, with eyes closed and “yaw” head movement). Greater activation of the left inferior parietal lobule and less deactivation of the left vestibular nucleus were correlated with smaller ellipse area (i.e., less body sway) (Figure 6A). The scatterplot of correlation between IPL activity and balance is also included for illustration purposes (Figure 6B). Left side tap-induced brain activity was also negatively correlated with body sway in Romberg stance on firm surface, with eyes closed and “pitch” head movement: Less deactivation of left cerebellum (lobule VI and VIIIB), and bilateral vestibular nuclei were correlated with smaller ellipse area (Figure 6C). This is further illustrated by a scatterplot to illuminate the direction of correlation (Figure 6D).

**Table 6:** **Results of correlations between tap-induced brain activity and ellipse area**.

***t*-contrast**	**Brain region**	**MNI coordinates *x,y,z***	**Cluster size**	***t*-value**	***P*-value**	**β (CI)**
**LEFT TAP AND ELLIPSE AREA (ROMBERG,EYES CLOSED,YAW)**						
Positive	No suprathreshold voxels were found					
Negative	Left Inferior Parietal Lobule	−58, −26, 26	16	8.09	0.0001	0.25 (±0.18)
	Left Vestibular Nucleus	−10, −32, −35	28	4.24	0.0010^*^	−0.02 (±0.02)
**LEFT TAP AND ELLIPSE AREA (ROMBERG,EYES CLOSED,PITCH)**						
Positive	No suprathreshold voxels were found					
Negative	Right Vestibular Nucleus	24, −38, −37	93	6.16	0.0001	−0.09 (±0.05)
	Left Cerebellar Lobule VI	−36, −44, −31	75	6.07	0.0001	−0.15 (±0.05)
	Left Vestibular Nucleus	−26, −32, −21	56	5.66	0.0001	−0.13 (±0.07)
	Left Cerebellar Lobule VIIIB	−2, −61, −35	23	5.13	0.0001	−0.07 (±0.06)

* *Significant at FWE. P < 0.05. Mean beta values (β) are represented with %95 confidence interval (CI)*.

**Figure 7:**
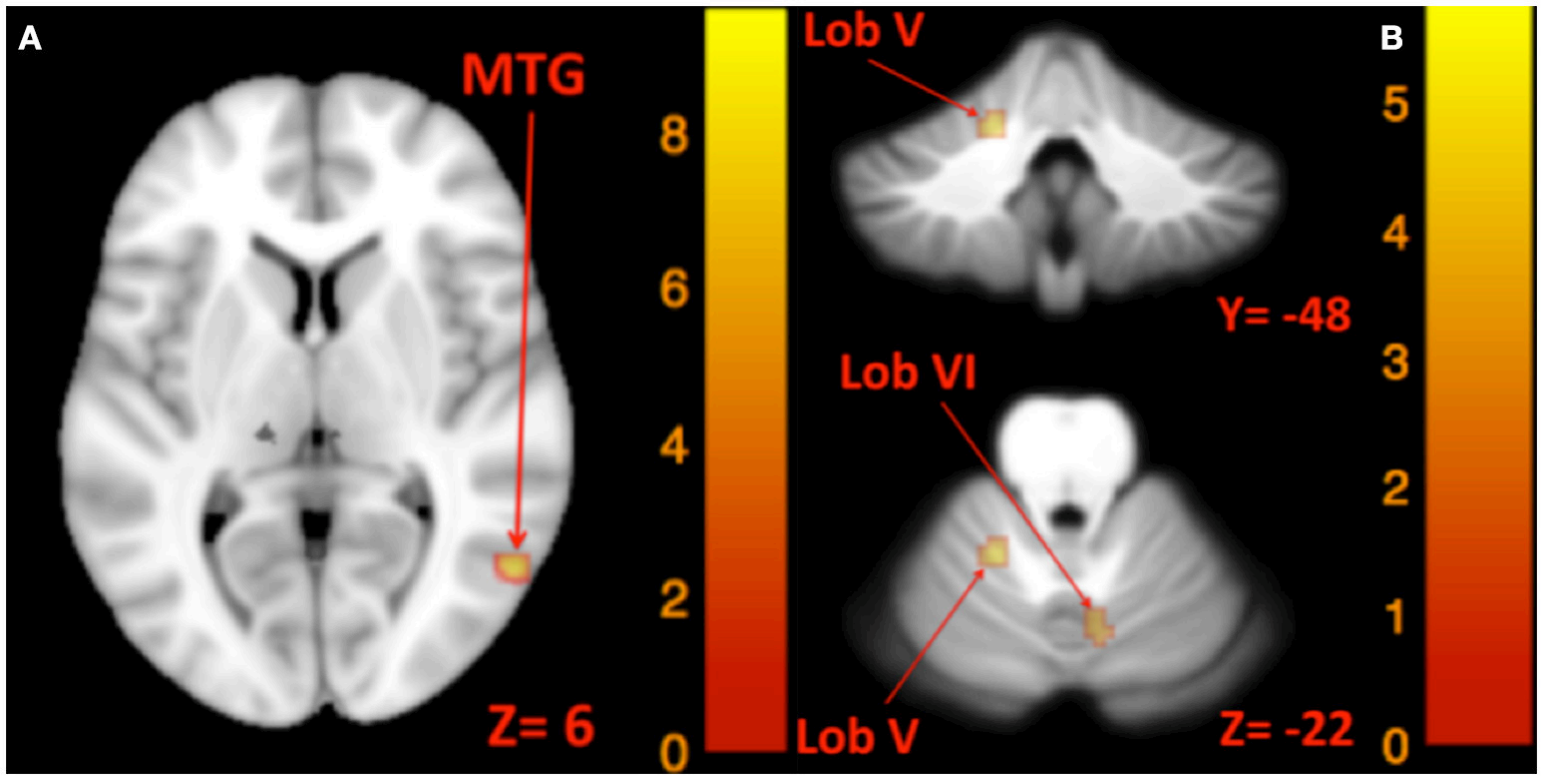
**Map of correlation between ellipse area and brain activity induced by left and right tone**. Left tone correlation with ellipse area in Romberg stance on firm surface, eyes closed, yaw head movement **(A)**; Right tone correlation with ellipse area in Romberg stance on firm surface, eyes closed, yaw head movement **(B)**. The corresponding *t*-value is presented next to each figure. Left and right sides refer to the left and right sides of the brain, respectively. N.S, Non-Significant; MTG, Middle Temporal Gyrus.

The results for correlation between tone-induced brain activity and ellipse area are shown in Table 7. The left side tone-induced brain activity was correlated with balance (in Romberg stance on firm surface, with eyes closed and yaw head movement), as greater activation in right middle temporal gyrus was associated with smaller ellipse area and better balance control (Figure 7A). Moreover, higher activation in the right cerebellum (lobule VI), right vestibular nucleus, and left cerebellum (lobule V) during the right side tones was associated with smaller ellipse area during Romberg stance on firm surface, with eyes closed and yaw head movements (Figure 7B).

**Table 7:** **Results of correlations between tone-induced brain activity and ellipse area**.

***t*-contrast**	**Brain region**	**MNI coordinates *x,y,z***	**Cluster size**	***t*-value**	***P*-value**	**β (CI)**
**LEFT TONE AND ELLIPSE AREA (ROMBERG,EYES CLOSED,YAW)**						
Positive	No suprathreshold voxels were found					
Negative	Right Middle Temporal Gyrus	56, −62, 6	19	9.65	0.0001	−0.05 (±0.19)
**RIGHT TONE AND ELLIPSE AREA (ROMBERG,EYES CLOSED,YAW)**						
Positive	No suprathreshold voxels were found					
Negative	Right Vestibular Nucleus	22, −36, −23	32	4.19	0.0020^*^	0.02 (±0.06)
	Right Cerebellar Lobule VI	8, −68, −25	37	5.19	0.0001	−0.01 (±0.05)
	Left Cerebellar Lobule V	−16, −48, −23	17	5.30	0.0001	−0.02 (±0.07)

* *Significant at FWE. P < 0.05. Mean beta values (β) are represented with %95 confidence interval (CI)*.

**Figure 8:**
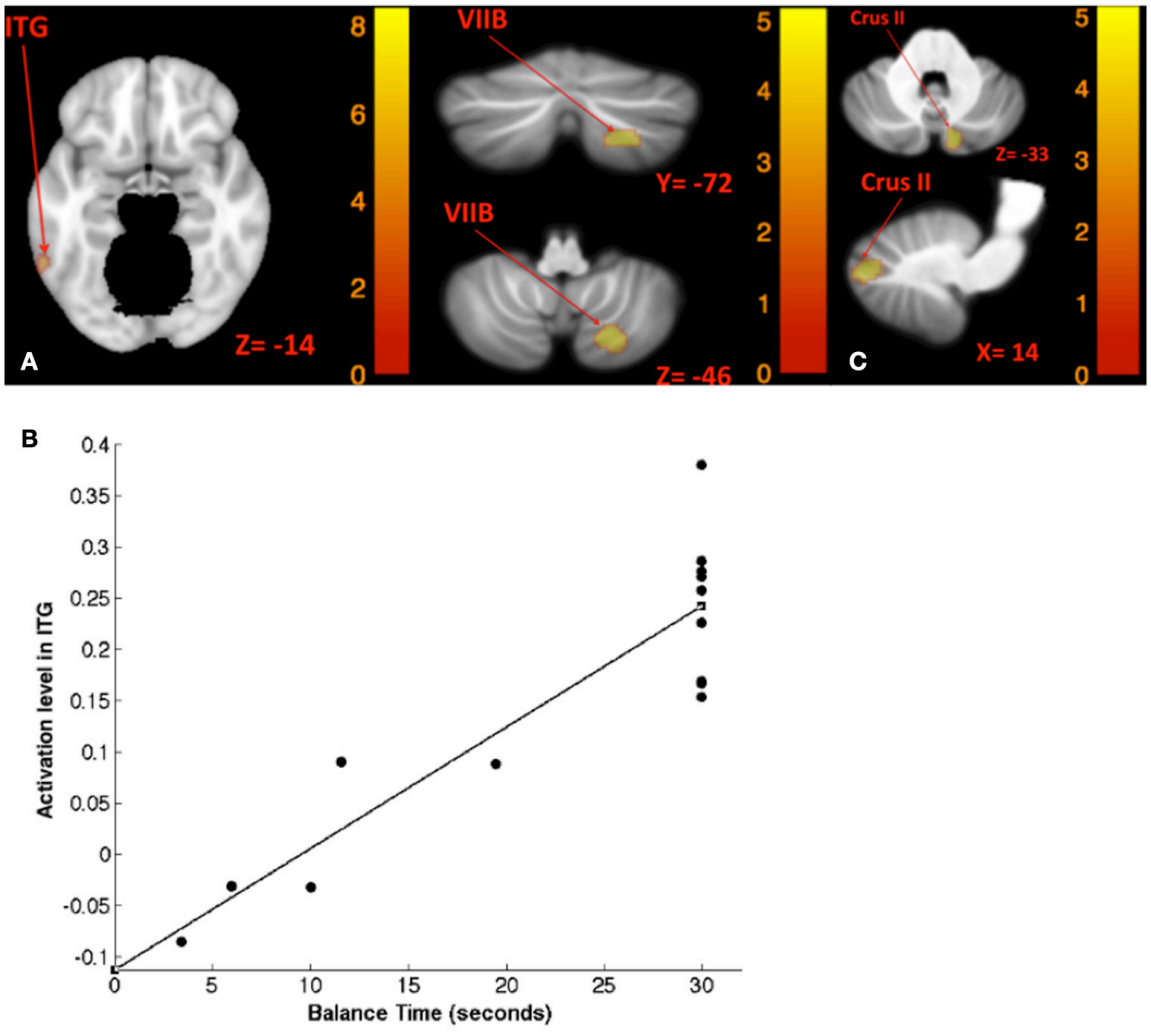
**Map of correlation between balance time and brain activity induced by left tone stimulation**. Left tone correlation with balance time in Tandem stance on compliant surface, eyes open, yaw head movement **(A)**; the scatter plot illustrates the correlation between balance time and activation in ITG suggesting that greater activation of ITG is correlated with lengthier balance time **(B)**; left tone correlation with balance time in Tandem stance on compliant surface, eyes open, pitch head movement **(C)**. The corresponding *t*-value is presented next to each figure. Left and right sides refer to the left and right sides of the brain, respectively. N.S, Non-Significant; ITG, Inferior Temporal gyrus.

Table 8 shows the results for correlation between tone-induced brain activity and balance maintenance time (i.e., time to step out). Greater activation in left inferior temporal gyrus and right cerebellum (lobule VIIB) were associated with longer balance maintenance time in tandem stance on compliant surface with “yaw” head movement (Figures 8A,B). Also, greater activation in right cerebellum (Crus II) in response to left tone was associated with longer balance maintenance time in tandem stance on compliant surface with “pitch” head movement (Figure 8C).

**Table 8:** **Results of correlations between tone-induced brain activity and balance time**.

***t*-contrast**	**Brain region**	**MNI coordinates x,y,z**	**Cluster size**	***t*-value**	***P*-value**	**β (CI)**
**LEFT TONE AND BALANCE TIME (TANDEM ON FOAM, YAW)**						
Positive	Left Inferior Temporal Gyrus	−62, −50, −14	20	7.83	0.0001	0.15 (±.07)
	Right Cerebellar Lobule VIIB	22, −72, −47	73	5.20	0.0001	0.08 (±.05)
Negative	No suprathreshold voxels were found					
**LEFT TONE AND BALANCE TIME (TANDEM ON FOAM, PITCH)**						
Positive	Right Cerebellar Crus II	14, −82, −33	87	5.18	0.0001	0.04 (±.03)
Negative	No suprathreshold voxels were found					

*Mean beta values (β) are represented with %95 confidence interval (CI)*.

#### Left tap vs. right tap

No suprathreshold voxels showed a significant difference between left and right side taps (Table 9).

**Table 9:** **Results of comparison between left and right side brain activity elicited by tap and tone stimuli**.

***t*-contrast**	**Brain region**	**MNI coordinates x,y,z**	**Cluster size**	***t*-value**	***P*-value**	**β (CI)**
**LEFT TAP VS. RIGHT TAP**						
Left tap > right tap	No suprathreshold voxels were found					
Left tap < right tap	No suprathreshold voxels were found					
**LEFT TONE VS. RIGHT TONE**						
Left tone > right tone	No suprathreshold voxels were found					
Left tone < right tone	Left Postcentral Gyrus	−62, −22, 14	33	6.56	0.0001	0.38 (±0.28)
	Right Temporal Lobe, Sub-Gyral	42, −24, 0	23	6.21	0.0001	0.18 (±0.15)
**LEFT TONE VS. BOTH SIDES TONE**						
Left tone > both tone	No suprathreshold voxels were found					
Left tone < both tone	Left Superior Temporal Gyrus	−42, −40, 10	20	7.52	0.0001	0.05 (±0.06)
**RIGHT TONE VS. BOTH SIDES TONE**						
Right tone >both tone	No suprathreshold voxels were found					
Right tone < both tone	Right Anterior Cingulate	8, 12, 26	15	7.94	0.0001	0.005 (±0.03)
	Right Precuneus	12, −68, 44	23	6.89	0.0001	−0.04 (±0.10)
	Right Superior Temporal Gyrus	64, −18, −0	51	6.30	0.0001	0.06 (±0.12)
	Left Superior Temporal Gyrus	−62, −24, 8	41	5.65	0.0001	0.20 (±0.21)

*Mean beta values (β) are represented with %95 confidence interval (CI)*.

#### Left tone vs. right tone

**Figure 9:**
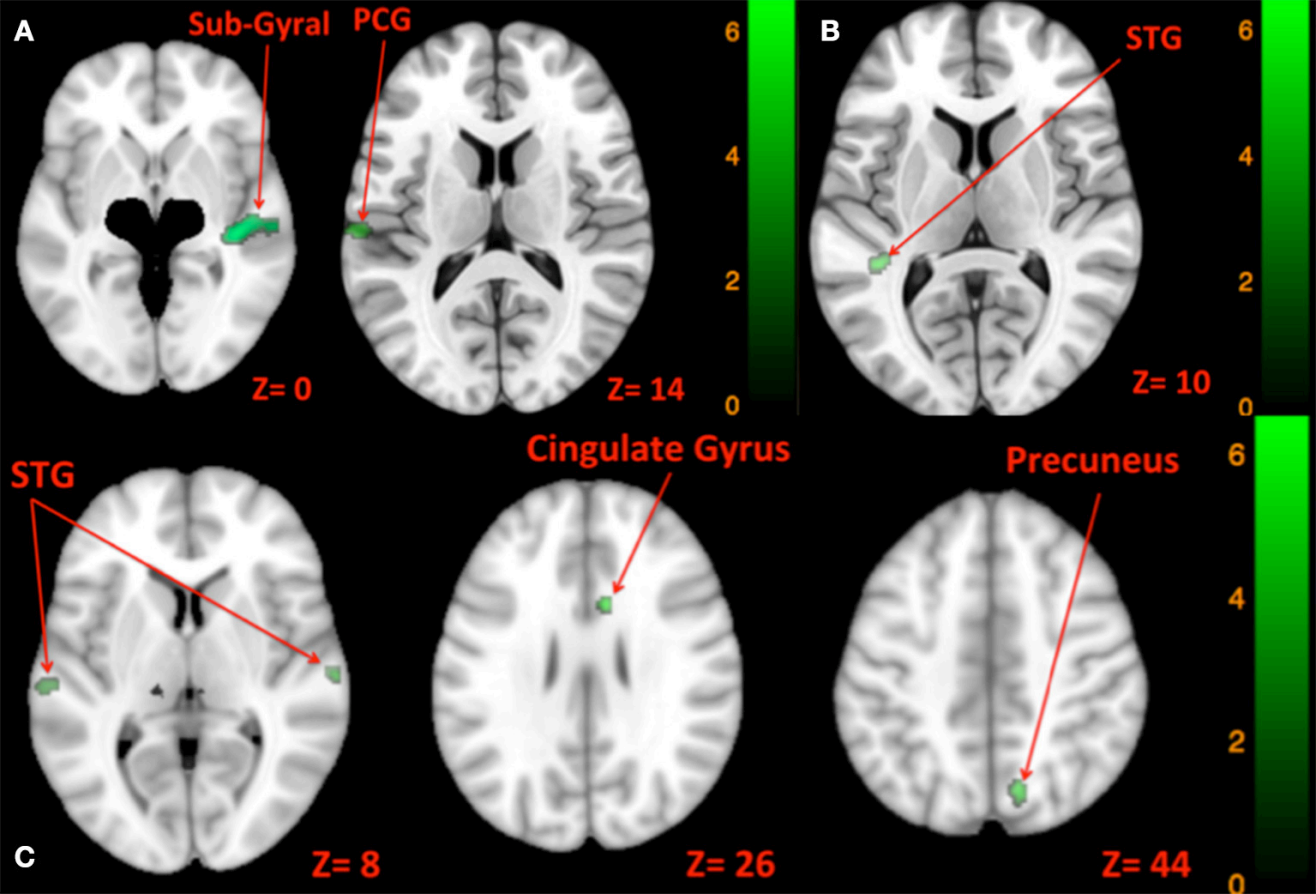
**Map of comparison between brain activity elicited by right vs. left side tone [A:** Left tone > Right tone (N.S); Left tone < Right tone (green)], left vs. both sides tone [**B**: Left tone > Both side tone (N.S); Left tone < Both side tone (green)], and right vs. both sides tone [**C**: Right tone > Both side tone (N.S); Right tone < Both side tone (green)]. The corresponding *t*-value is presented next to each figure. Left and right sides refer to the left and right sides of the brain, respectively. N.S, Non-Significant; PCG, Post Central Gyrus; STG, Superior Temporal Gyrus.

Right tone resulted in greater activation of the right temporal lobe sub-gyral and left postcentral gyrus than left side tone stimulation (Table 9, Figure 9A).

#### Left tone vs. both sides tone

There was greater activation of left STG with tone stimulation delivered to both sides at 90 dB SPL than to the left side at 130 dB SPL (Table 9, Figure 9B).

#### Right tone vs. both sides tone

There was greater activation of bilateral superior temporal gyri, right precuneus, and right anterior cingulate gyrus with tone stimulation delivered to both sides at 90 dB SPL than to the right side at 130 dB SPL (Table 9, Figure 9C).

### Skull tap vs. auditory tone burst stimulation: subjects' perceptions

The majority of subjects found the auditory tone bursts loud and unpleasant. One subject withdrew from the study specifically due to the loudness of the auditory tone bursts. Although we provided padding around the ears to help muffle the scanner noise, the fact that subjects did not have the benefit of earplugs while wearing the headphones inside the scanner was an additional source of discomfort for auditory trials. In some cases subjects had a hard time discerning the auditory tone bursts from the scanner noise, whereas every single subject reported feeling the taps. Subjects reported no discomfort with the skull tap stimulation.

## Discussion

Here we provide the first evidence of using skull taps to elicit vestibular fMRI activity. Conjunction analyses revealed that skull taps elicit overlapping activation patterns with auditory tone bursts (i.e., STG), and both modes of stimulation activate previously identified regions of the vestibular network (Lopez et al., 2012; zu Eulenburg et al., 2012; Kirsch et al., 2015). Additionally, we found that skull taps elicit more robust activity compared to auditory tone bursts, with brain activation from taps frequently surviving family-wise error corrections. The taps were also better tolerated by subjects than the auditory tones, further supporting their potential use in future clinical and basic science research.

We also provided evidence that individual differences in amplitude of activation and deactivation in vestibular regions in response to vestibular stimulation are associated with better balance and postural control. To our knowledge, the correlation of vestibular brain activity with balance has been addressed only in a few recent fNIRS studies (Karim et al., 2012; Huppert et al., 2013). We showed that not only the quality of balance (indicated by the amount of body sway) but also the ability to maintain balance for a longer time (indicated by the balance time) depends on individuals' brain activation levels, particularly in vestibular cortical regions and the vestibular nuclei. Thus, vestibular brain activity could potentially serve as a predictor of individual differences in susceptibility to falling, and could further be used to index neuroplasticity occurring with balance interventions.

### Vestibular cortex response elicited by skull taps and auditory tone bursts

Although there were some discrepancies between the activation patterns elicited by the auditory tone burst vs. the skull-tap, these weighed in favor of the skull-tap method being a more specific way of stimulating the vestibular network. While skull tap resulted in consistent activation of the right STG and the bilateral insula—areas identified in meta analyses of vestibular cortical activity (zu Eulenburg et al., 2012; Lopez et al., 2012)—the left side auditory tone burst stimulation only activated the right STG. Right side auditory stimulation did not elicit any significant activation. Moreover, the left side auditory tone burst resulted in a less vestibular-specific activity pattern, activating somatosensory cortices, the frontal gyrus and the precentral gyrus. This could be partly related to the noisier processing of auditory tone bursts over the scanner noise. Considering that subjects had to wear the headphones instead of earplugs inside the scanner the tones could have been difficult to detect. Also, the potential aversive effects of high decibel stimulation could be another source of inconsistency in tone-induced responses.

Interestingly, the skull tap predominantly deactivated the cerebellar lobules VI and VIIIA, along with cortical regions involved in somatosensory processing (e.g., frontal and parietal cortices) and subcortical regions such as thalamus, which relays information between vestibular nuclei and cortex. This may reflect a shift in attention toward vestibular processing and away from other sensory modalities. Similar deactivation of somatosensory and visual cortices has also been reported during vestibular stimulation by Schlindwein et al. (2008). Skull tap also resulted in deactivation of the contralateral vestibular nucleus, which replicates a known inhibitory projection reported in the animal literature (Shimazu and Precht, 1966) and recently documented in human subjects with diffusion tractography MRI (Kirsch et al., 2015). Also, the deactivation of vestibular nuclei has shown to be related to resolving state estimation errors. Brooks et al. (2015) have shown that when there is a mismatch between predicted and actual sensory consequences, the fastigial nuclei's projection to vestibular neurons activates reflex pathways to maintain postural control. However, both the fastigial and vestibular nuclei are generally suppressed during voluntary movement in which efference copy is used to suppress state estimation errors. In our case, we delivered vestibular stimulation in the absence of movement, potentially resulting in sensory conflict. Our finding of less deactivation of vestibular nuclei being correlated with better balance could therefore suggest that a better ability to maintain balance is associated with being better able to resolve prediction errors.

We also found that balance abilities correlated with activation in cerebellar lobules V, VI, and VIII, but only for balance tasks that were performed with eyes closed (i.e., more vestibular reliant). Similar cerebellar regions have also been activated in previous studies using different modes of vestibular stimulation; for instance, auditory short tone burst (Schlindwein et al., 2008) and galvanic vestibular stimulation (Stephan et al., 2005) altered the activation of cerebellar lobules VI, VIIIB, Crus I, Crus II, and the dentate nucleus.

### Laterality effects of stimulation modes

We compared the brain activation pattern elicited by left vs. right side stimulation for both auditory tone bursts and skull taps. There are some inconsistencies in the literature regarding the laterality of VEMPs. Although these inconsistencies can be partly explained considering the location of VEMP responses [i.e., oVEMPs show the vestibular processing in crossed otolith-ocular pathways, whereas cVEMP reflects the function of uncrossed otolith-spinal pathways (Iwasaki et al., 2007)] and different electrode setups (Ertl et al., 2015), different modes of stimulation have shown to be the main driving factor for different patterns of laterality in VEMPs. We observed that skull tap stimulation resulted in bilateral vestibular cortical activation regardless of the side of stimulation, but specific contralateral deactivation of the vestibular nucleus. Brantberg and colleagues also showed that skull taps elicit bilateral cVEMPs, with a net excitatory response on the ipsilateral side (Brantberg and Tribukait, 2002) and a net inhibitory on the contralateral side (Brantberg et al., 2009). Our finding that skull tap prominently deactivated the contralateral vestibular nucleus is compatible with this previously reported pattern of cVEMPs. Brantberg et al. (2009) suggested that skull taps induce this bilateral response possibly through vibration (more ipsilateral) and translation (more contralateral) mechanisms (Brantberg et al., 2009). The bilaterality of tap-induced cVEMPs is independent of recording location (Brantberg et al., 2009) and it has been confirmed by oVEMP measurements as well (Holmeslet et al., 2011).

As for the auditory tone bursts, our results support a laterality of vestibular processing. Murofushi and colleagues found a dominant ipsilateral cVEMP using short tone bursts and auditory clicks (Murofushi et al., 2004). Likewise, Schlindwein et al. reported a laterality effect using short tone bursts and acoustic stimuli in an fMRI experiment: While the overall brain activation pattern was bilateral, tone burst-induced vestibular processing was predominantly ipsilateral and in the right hemisphere, whereas the lower decibel acoustic stimulus resulted in a contralateral response, more on the left hemisphere (Schlindwein et al., 2008). Our findings also showed a left hemispheric activation pattern resulting from lower decibel acoustic stimulation (Table 3: Both sides tone, 90 dB vs. rest). However, our results for tone-induced brain activation do not entirely overlap with the Schlindwein et al. findings: While left tone predominantly activated the ipsilateral hemisphere (Table 3: Left tone 130 dB vs. rest), the right tone predominantly deactivated the ipsilateral hemisphere (Table 3: Right tone 130 dB vs. rest). Nevertheless, our results support that auditory tone bursts results in a lateralized vestibular evoked response.

The conjunction analyses also revealed a laterality effect of auditory tone burst stimulation; the commonly activated regions by left side tap and left side tone were bilateral STG, and the commonly activated region by right side tap and right side tone was left STG. The further confirmation for the laterality effects came from the results of conjunction between the two stimulation modes in opposite sides: The commonly activated region by left side tap and right side tone was the left STG; whereas the right side tap and left side tone commonly activated the right STG and left insula. The opposite hemispheric effect seems to be mainly related to right auditory tone bursts, because skull taps elicited bilateral vestibular activation and left auditory tone bursts also evoked weak bilateral responses. One possible mechanism could be that tap-induced vibration engages different pathways to travel within the vestibular system than auditory tone bursts (e.g., the vibration caused by skull taps impacts utricular and saccular pathways, whereas tone bursts primarily engage the saccular structure Holmeslet et al., 2011).

### Correlation between vestibular cortex activity and balance

We observed a correlation between individual differences in balance control and brain activity elicited by both modes of stimulation; however, the correlation predominantly emerged with left side stimulation. This finding could potentially be related to the right hemispheric dominance of vestibular processing (Dieterich, 2003; Janzen et al., 2008). The correlation results suggested that those who exhibited greater vestibular activation in response to left side stimulation (either by skull taps or auditory tone bursts) had better balance control (i.e., less amount of body sway and longer balance stability).

Although balance performance reflects a multisensory integration of visual, vestibular, and proprioceptive signals, we enforced greater reliance on vestibular processing by removing visual input and adding head movements. Thus, performance in these tasks mainly represents vestibularly mediated balance control, which we found to be correlated with vestibular brain activity.

The balance time correlation was only evident with auditory tone burst stimulation, and not the skull taps. More specifically, the positive correlation between cerebellar activity and balance time emerged in lobule VIIB and Crus II, which have been previously linked to spatial processing (Stoodley et al., 2012). One potential reason for the correlation between balance maintenance time and tone stimulation, but not tap stimulation, could be related to the choice of balance task as the covariate. As explained in the results section, we used “tandem stance on compliant surface with yaw/pitch head movement” to assess the balance time correlation with vestibular activity. In this task the visual input was not removed; therefore, there was less dependency on vestibular function compared to “Romberg stance with eyes closed and yaw/pitch head movement,” in which there was no visual input. As discussed earlier, our findings suggest that skull taps elicit a more vestibular-specific activation compared to auditory tone bursts. This could potentially explain why brain activation elicited by skull tap was correlated with balance when performance was more vestibularly mediated (i.e., performed with eyes closed); but it failed to show any correlation with balance when the balance performance was less reliant on vestibular inputs (i.e., performed with eyes open).

Overall, these correlations suggest that those with greater vestibular cortex excitability or more efficient transmission within vestibular networks have better balance control. Although the left and right vestibular nuclei were the only clusters surviving the family-wise error correction (FEW) for multiple comparisons in correlation analyses, the remaining clusters fit the previously identified vestibular network in studies of balance assessment in upright stance using fNIRS (Karim H. et al., 2013). This provides further validation for associating upright balance performance with vestibular activity elicited in a supine position.

### Validity of vestibular evoked activation inside scanner

We included the oVEMP assessments to address the validity of our stimulation inside the scanner. Our findings showed that subjects exhibited typical oVEMP characteristics in response to the skull tap and auditory tone burst stimulation outside the scanner. This supports the notion that the brain activation elicited by the same stimulation modes inside the scanner can be interpreted as vestibular signal processing.

### Subjective comfort

Based on the anecdotal reports in our sample of 14 subjects, the MR compatible skull tap is well-tolerated inside the scanner, whereas auditory tone bursts cause discomfort and distress, similar to what has been previously reported (Wackym et al., 2012). Therefore, using the skull tap stimulation minimizes the potential artifacts of aversive brain activation elicited by auditory tone bursts.

## Limitations

The skull taps are perceived by subjects on the facial skin, which could result in somatosensory processing. We did not observe activation in the somatosensory cortex during taps, however; instead the responses were predominately in regions that have been previously linked to vestibular processing. Nevertheless, future studies would benefit from implementing a tactile stimulation control condition.

## Conclusion

In sum, we found that the skull tap stimulation results in activation of canonical vestibular cortex as well as cerebellar and brainstem regions known to process vestibular inputs. This supports the skull tap as an effective method for studying human vestibular processing, especially in otolithic pathology. This is of high importance in longitudinal experiments, in which subjects' comfort is essential for minimizing the aversive effects and maintaining enrollment. Further, we provided evidence of the association between quantitative measures of balance control and vestibular brain activation.

## Ethics statement

This study was carried out in accordance with the recommendations of University of Michigan Medical Institutional Review Board with written informed consent from all subjects. The protocol was approved by the University of Michigan Medical Institutional Review Board.

## Author contributions

RS, AM, JB, and SW designed the experiment. IK and YD engineered the devices. CK and FN conducted the experiment and analyzed the data. All authors wrote the manuscript.

### Conflict of interest statement

The authors declare that the research was conducted in the absence of any commercial or financial relationships that could be construed as a potential conflict of interest.

## Supplementary material

Supplementary Material Figure 1 **The image represents MR compatible Pneumatic Tactile Pulse System (manufactured by Engineering Acoustics Inc.), and the way it's placed over subject's cheekbones**.
