# Supplementary figures and images for: Functional Brain Activation in Response to a Clinical Vestibular Test Correlates with Balance
Source: Front Syst Neurosci. 2017 Mar 10;11:11. doi: 10.3389/fnsys.2017.00011 (PMC5344901; doi:10.3389/fnsys.2017.00011)

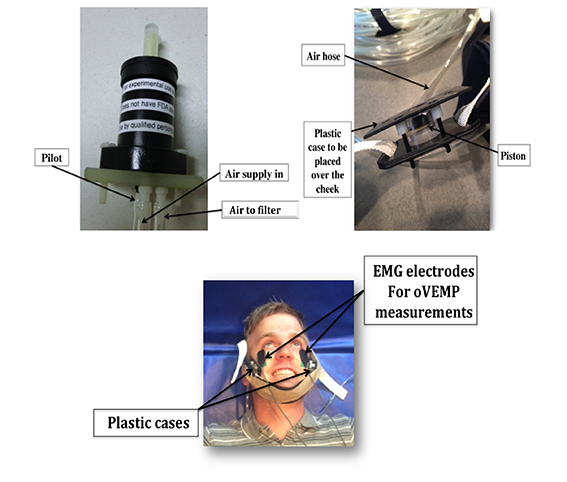

Supplement: Supplementary Material Figure 1 — The image represents MR compatible Pneumatic Tactile Pulse System (manufactured by Engineering Acoustics Inc.), and the way it's placed over subject's cheekbones. [file Image1.TIF]
